# Impact on Oral Health‐Related Quality of Life in Skeletal Class III Patients Treated With Orthognathic Surgery‐First Approach Using Orthodontic Aligners or Fixed Appliances: A Controlled Clinical Study

**DOI:** 10.1111/ocr.70054

**Published:** 2025-11-07

**Authors:** Arthur Cunha, Luana Karine Amaro Silva, Henrique Martins da Silveira, José Augusto Mendes Miguel

**Affiliations:** ^1^ Clinic of Orthodontics State University of Rio de Janeiro—UERJ Rio de Janeiro Brazil; ^2^ Oral and Maxillofacial Surgery Division Pedro Ernesto University Hospital, State University of Rio de Janeiro Rio de Janeiro Brazil

**Keywords:** clear aligners, orthodontic appliances, removable, orthognathic surgery, surgery‐first approach

## Abstract

**Objective:**

This prospective study aimed to evaluate the impact on oral health‐related quality of life (OHRQoL) in skeletal Class III patients treated with the surgery‐first approach using either orthodontic aligners or fixed appliances.

**Materials and Methods:**

Patients were treated using the Surgery‐First Approach (SFA) combined with either clear aligner (CA) or conventional fixed appliances (FA). A total of 20 patients (male and female) were allocated to the CA group, while 14 were included in the FA group. OHRQoL was assessed using the Orthognathic Quality of Life Questionnaire (OQLQ), the Oral Health Impact Profile‐14 (OHIP‐14) and the SF‐36 at different time periods: (T0) pre‐treatment, (T1) 1 week after orthodontic appliance installation, (T2) 4 weeks post‐surgery and (T3) 6 months post‐surgery. Data analysis was conducted using the Friedman and Mann–Whitney tests, with a significance level of 5%.

**Results:**

No statistically significant differences were found in OHRQoL scores between the CA and FA groups at baseline and after 6 months of treatment. However, significant intra‐group improvements were observed over time, particularly between the first and sixth months of treatment.

**Conclusions:**

Both ortho‐surgical approaches, using either clear aligners or fixed appliances, resulted in significant improvements in patient quality of life. These findings highlight the positive impact of orthognathic treatment on dentofacial correction and overall well‐being, regardless of the appliance type.

**Trail Registration:**

ClinicalTrials.gov NCT05822271.

## 1 Introduction

The demand for faster and more aesthetic treatments has made therapy with clear aligner (CA) more common, with their indications expanded to treat more severe malocclusions, including surgical cases [1, 2]. Initially, CA was advocated to correct minor tooth positioning irregularities [3]. Nowadays, patients are starting aligner therapy for orthodontic‐surgical treatment, despite the limited and controversial level of scientific evidence [4]. Particularly, the literature supporting the use of aligners in conjunction with orthognathic consists predominantly of case reports and expert opinions [1]. It is important to note that these studies provide minor emphasis on the improvement of procedures in quality of life (QoL) and patient perception of these benefits.

Oral health‐related quality of life (OHRQoL) is defined as a multidimensional concept that reflects people's comfort while eating, sleeping, self‐esteem and social interaction related to oral health [5]. Recent studies have highlighted the relationship between malocclusion and OHRQoL, focusing on understanding patient experiences in the treatment of severe dentofacial deformities [6], and, consequently, in orthodontic care [7]. It is well‐known that malocclusion negatively impacts oral function and the psychosocial aspects of orthodontic patients [8]. Moreover, orthodontic treatment inevitably attends some adverse effects such as pain, anxiety and deterioration of OHRQoL [9].

Although fixed appliances (FA) are effective for treating various malocclusions, they may negatively impact QoL [10] due to discomfort, pain and functional limitations after treatment begins [11, 12]. In contrast, CA due to their aesthetic and structural features, may reduce these adverse effects. A systematic review reported better OHRQoL and lower pain levels in patients treated with CA compared to FA [13]. Additionally, some studies suggest that patients are more psychologically affected by the appearance of orthodontic appliances than by the severity of their malocclusion [14]. Nevertheless, reports directly comparing CA and FA remain limited, especially in surgical cases, making it difficult to determine which appliance offers better QoL [15]. A recent study using the surgery‐first approach (SFA) reported better scores in the Orthognathic Quality of Life Questionnaire (OQLQ) and in Oral Health Impact Profile—Short Version (OHIP‐14) with CA [16]. However, systematic reviews have yet to find conclusive differences between aligners and FA in terms of OHRQoL [17], and no studies have directly compared both modalities specifically within the context of orthognathic surgery.

Given the currently deficient evidence, further researches are needed to evaluate the QoL associated with CA in conjunction with orthognathic surgery. Expanding studies in this area will provide deeper insights into patients' experiences, enabling the refinement of treatment protocols to optimise both functional and aesthetic outcomes. Understanding patient experiences can encourage the adoption of more comprehensive and patient‐centered practices, ensuring that their expectations and well‐being are prioritised during treatment. Consequently, the aim of this study was to assess the impact of OHRQoL in treatment with CA and FA combined with orthognathic surgery in correcting skeletal discrepancies.

## 2 Materials and Methods

**FIGURE 1:**
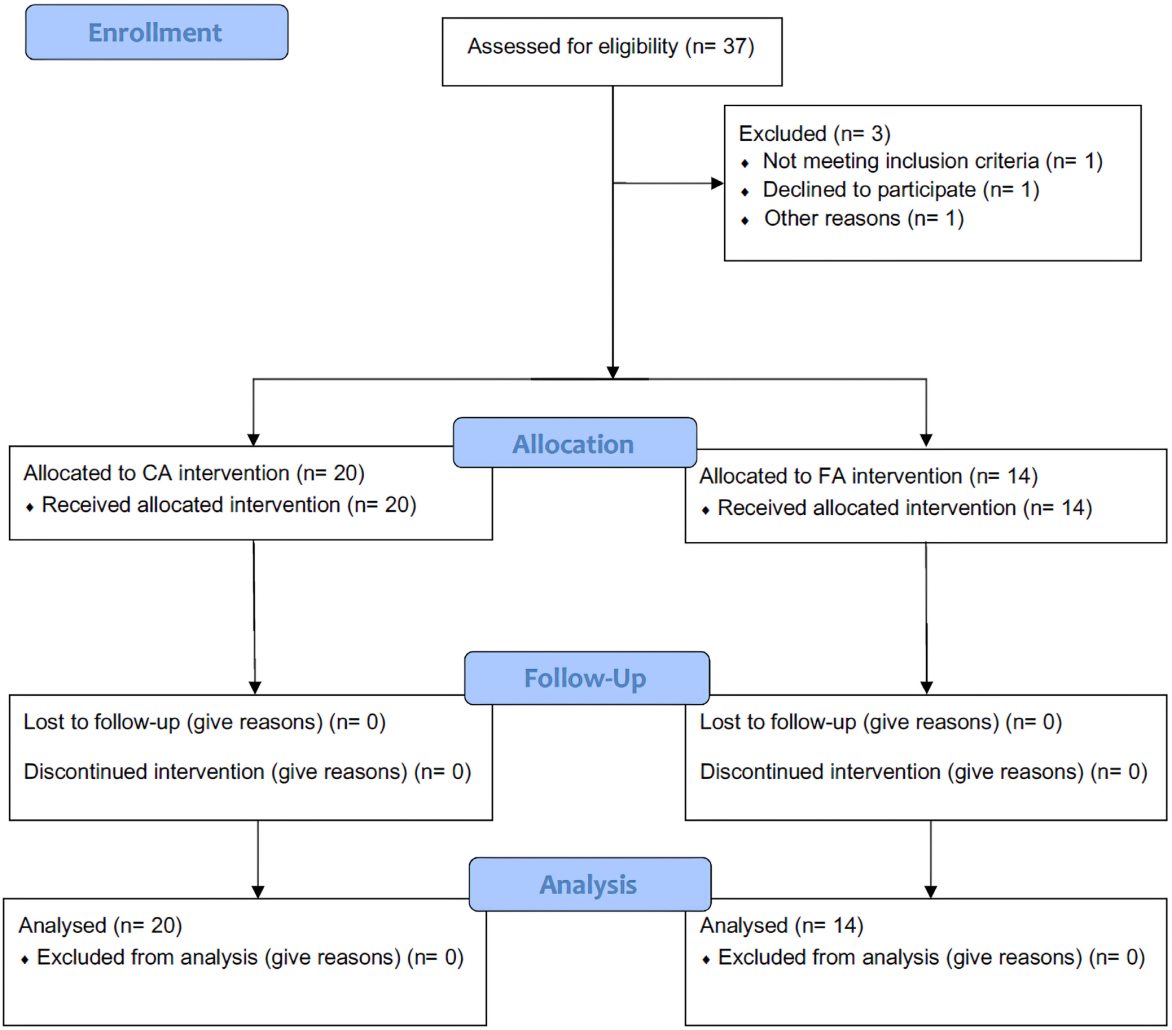
CONSORT flow diagram illustrating patient enrollment, allocation, follow‐up and analysis. CA = clear aligner, FA = fixed appliance.

This controlled clinical trial was registered on ClinicalTrials.gov (NCT05822271) and in the Brazilian Registry of Clinical Trials (REBEC). The study was approved by the Research Ethics Committee of Pedro Ernesto University Hospital, affiliated with the State University of Rio de Janeiro (UERJ), under protocol number (CEP/HUPE: 51444621.0.0000.5259). The sample size calculation was performed using the Harvard sample size calculator (http://hedwig.mgh.harvard.edu/sample_size/js/js_associative_quant.html). The primary outcome was the difference in OHRQoL scores between groups, based on a previous clinical trial [16], a minimum clinically significant difference of 12 points was adopted as the expected effect size. A standard deviation of 10.5 was used. Assuming a two‐sided test, an alpha level of 0.05, and a power of 80%, the estimated sample size required was 14 subjects per group. Patients were prospectively enrolled and allocated into treatment groups based on a consecutive inclusion strategy. Due to practical and ethical considerations related to treatment availability and the real‐world clinical setting. Initially, patients meeting the eligibility criteria for treatment with clear aligners were allocated to the CA group. After reaching the minimum required sample size for this group, subsequent eligible patients were assigned to the FA group. Among the participants, three women from the CA group were excluded from the study for different reasons: relocation, cooperation difficulties with the treatment, and lack of interest in starting treatment (Figure 1).

The inclusion criteria were: patients with no potential growth (stage CS6 of cervical vertebral maturation); diagnosis of skeletal Class III by negative Wits assessment; aetiology related to mandibular excess, maxillary deficiency, or a combination of both, requiring bimaxillary surgery. Exclusion criteria included: presence of any type of craniofacial anomaly or prior fracture of facial bones; issues related to the temporomandibular joint; periodontal problems affecting dental compensations; patients requiring tooth extractions (except third molars); curve of Spee greater than 6 mm; mandibular asymmetries greater than 6 mm; use of any medication that contraindicated surgical treatment.

Digital models of the patients and the DICOM file were imported into Dolphin Imaging software, version 11.95 Premium (Dolphin Imaging, Chatsworth, CA, USA), for virtual surgical planning (VSP). Subsequently, the patients underwent facial analysis and orthodontic surgical planning relevant to the SFA protocol used by the researchers of this study [18]. The study period extended from August 2022 to 2024, beginning with the first surgical procedures and concluding with the 6‐month follow‐up evaluations of all included patients. All patients underwent surgery at the Pedro Ernesto University Hospital (HUPE), performed by a professor of Oral and Maxillofacial Surgery and Traumatology (H.M.S). The bimaxillary surgical protocol included LeFort 1 osteotomy and bilateral sagittal split osteotomy (BSSO), and preoperative, intraoperative and postoperative care were standardised for all patients in both groups. Six male patients in the CA group and five in the FA group (2 female and 4 male) underwent genioplasty. None of the patients underwent surgical segmentation of the maxilla.

Orthodontic treatments were carried out at the State University of Rio de Janeiro (UERJ), under the responsibility of the researcher (A.C) with supervision by the professor in charge of this research (J.A.M.). Complete orthodontic records were collected for both groups, including clinical examination, anamnesis, photographic documentation, digital dental models, panoramic radiograph, lateral cephalometric radiograph, intraoral scans and cone‐beam computed tomography (CBCT). Orthodontic appliances were installed between 1 and 2 weeks prior to surgery in both groups. In the CA group, patients' arches were previously scanned and the models were uploaded to the ClinCheck platform, Invisalign (Invisalign—Align Technology, San Jose, Calif, USA). In the first 10 aligners, cutouts were planned in the devices for the installation of buttons from molars to canines in both arches, aiming to maximise trans‐ and post‐surgical stability.

In the FA group, patients also had their arches previously scanned, but the models were uploaded to the NemoCast platform (NemoStudio, Leganés, Madrid, Spain). For treatment, pre‐adjusted 0.022″/0.028″ Roth prescription brackets were installed for alignment and levelling, incisor decompensation, arch coordination and occlusal refinement. All patients in the FA group had their treatments carried out with the same type of stainless‐steel FA, using wires and accessories from the same commercial brand (American Orthodontics, Sheboygan, WI, USA). Prior to surgery, 0.014″ annealed NiTi wires were inserted into the device so that no dental movements occurred, rendering the use of surgical guides unfeasible.

To ensure better trans‐surgical stabilisation control, intermaxillary fixation screws were used in both groups, assisting the use of surgical guides during the procedure. After orthognathic surgery, the surgical guide was installed to guide the jaw during this phase of post‐surgical occlusal instability, remaining in the oral cavity with elastics for 2–3 weeks. During this period, the guide was temporarily removed weekly for cleaning and occlusion evaluation.

The post‐surgical orthodontic phase initiated immediately following the use of surgical guides in both groups. In the CA group, after confirming occlusal stability by the use of the surgical guide, attachments were placed, and the initial active aligners were introduced with the support of light elastics to guide occlusion. During the first 3 months of treatment, aligners were changed every 5 days, and after this period, the interval was extended to the conventional 10‐day wear cycle. For attachment placement, Grandioso resin was used (VOCO GmbH, Cuxhaven, Germany). In the FA group, treatment began with active wires and light elastics to assist in occlusal guidance, facilitating the progressive alignment of teeth and ensuring stability of the planned orthodontic movements.

Data collection for the study was conducted at specific time points: (T0) before treatment; (T1) 1 week after orthodontic appliance installation; (T2) 4 weeks post‐surgery; and (T3) 6 months after surgery. The clinical examination started categorising patient aesthetics using the professional aesthetic perception of the IOTN‐AC (IOTN AC‐E), followed by the patient's self‐perceived aesthetics (IOTN AC‐A). Subsequently, patients' OHRQoL was assessed using pre‐established questionnaires: the OQLQ‐22 [19] in its Brazilian Portuguese validated version (B‐OQLQ) [20], the OHIP‐14 [21] also in its Brazilian Portuguese validated version [22], and the Medical Outcomes Study 36—Item Short‐Form Health Survey (SF‐36) [23], in its validated Brazilian Portuguese version [24]. Participants completed the questionnaires in a private location within the orthodontics department, taking about 30 min. A researcher (A.C) was present to clarify any doubts related to the assessments.

Data analysis was conducted using JAMOVI software (version 2.3.9, Sydney, Australia). Group compatibility was evaluated through the Wits appraisal, the Index of Orthodontic Treatment Complexity (IGC) and the IOTN AC‐E, applying the independent *t*‐test (continuous data) and the Mann–Whitney test (ordinal data). Examiner calibration for IGC assessment was ensured by randomly selecting 20 cases (10 from each group) for reassessment 1 week after the initial assessment. Weighted Kappa statistics were applied to assess examiner agreement for these cases. Intra‐examiner reliability was assessed by re‐evaluating the aesthetic index of the IOTN AC‐E for four individuals from each group 7 days after the initial evaluation (T0), and the Wilcoxon signed‐rank test was used to verify the consistency of the measurements. The Shapiro–Wilk test was used to check data normality. For statistical analysis, the Friedman test was applied to non‐parametric paired data, while the Mann–Whitney test was used for non‐parametric independent data, with a significance level set at 5%.

## 3 Results

Both treatment groups exhibited similar baseline characteristics. The CA group included 20 patients (11 females, 9 males) with a mean age of 23.3 years, while the FA group had 14 patients (5 females, 9 males) with a mean age of 19.9 years. Case severity, assessed by the Wits index, showed no significant differences between groups (−9.5 in CA vs. −8.2 in FA), nor did the IGC and IOTN AC‐E scores, reinforcing comparable initial conditions (*p* = 0.45, *p* = 0.83 and *p* = 0.25, respectively) (Table 1). Intra‐examiner reliability for the IGC evaluation demonstrated excellent agreement (0.943 in both groups) with high consistency (CI > 0.8, *p* < 0.001). No significant differences were observed in the IOTN AC‐E assessment (*p* > 0.05). The IOTN parameters showed significant improvements in both groups regarding the aesthetic component (IOTN‐AC). However, no significant differences were observed in IOTN classifications between aligners and fixed appliances, either at baseline or after 6 months of treatment (*p* > 0.05) (Table 2).

**TABLE 1:** Descriptive analysis of patients from clear aligners and fixed appliances groups.

		FA	CA	*p*
Sex	Female	5 (35%)	9 (45%)	0.58^a^
	Male	9 (65%)	11 (55%)	
Age ‐ mean (SD)		19.9 (2.8)	23.3 (6.2)	0.06^b^
Wits ‐ mean (SD)		−8.2 (5.5)	−9.5 (4.2)	0.45^b^
IGC ‐ mean (SD)		35.6 (17.1)	37.1 (14.6)	0.83^b^
IOTN AC ‐ E–mean (SD)		5.2 (2.4)	6.1 (2.3)	0.25^c^

Abbreviations: CA, clear aligners; FA, fixed appliance; IOTN AC ‐ E, professional aesthetic perception of the IOTN‐AC; SD, standard deviation.

^a^ Qui‐square test.

^b^ Independent *t*‐test.

^c^ Mann–Whitney *U*.

**TABLE 2:** Comparative statistics involving intra‐group IOTN, OQLQ and OHIP‐14 scores for clear aligners and fixed appliances groups.

	FA median (Q1–Q3)	CA median (Q1–Q3)	*p* ^b^
IOTN AC ‐ E T0	4 (4–7)	5.5 (5–8)	0.2
IOTN AC ‐ E T1	4 (4–6.2)	5 (3–7)	0.5
IOTN AC ‐ E T2	3 (2–4)	3 (2–4)	0.7
IOTN AC ‐ E T3	2 (2–2)	2 (1–2)	0.1
*p* ^a^	< 0.001	< 0.001	
IOTN AC ‐ A T0	4 (4–5.5)	5 (3.75–6)	0.4
IOTN AC ‐ A T1	4 (3–4)	4 (3–6)	0.3
IOTN AC ‐ A T2	2 (2–3.7)	3 (2–4)	0.2
IOTN AC ‐ A T3	2 (2–2)	2 (1–2)	0.2
*p* ^a^	< 0.001	< 0.001	
OQLO T0	55 (47.3–57)	61 (28–70)	0.7
OQLO T1	56,5 (44.3–69.8)	59 (28.8–71)	0.5
OQLO T2	33 (15.3–45.8)	26.5 (14–47.3)	0.8
OQLO T3	9 (0.5–18)	10.5 (2.7–24.3)	0.5
*p* ^a^	< 0.001	< 0.001	
OHIP‐14 T0	15.5 (10.5–20.8)	19 (6.7–30)	0.7
OHIP‐14 T1	15.5 (11.3–23)	17 (8.2–23)	0.9
OHIP‐14 T2	20.5 (8.2–27.8)	18 (9–24.5)	0.7
OHIP‐14 T3	9.5 (5.2–14.8)	8 (2.5–13)	0.6
*p* ^a^	0.1	0.02	

*Note:* (T0) pre‐treatment, (T1) 1 week after orthodontic appliance installation, (T2) 4 weeks post‐surgery and (T3) 6 months post‐surgery. *p* ≤ 0.05 = statistically significant.

Abbreviations: CA, clear aligners; FA, fixed appliance; IOTN AC ‐ A, self‐perception of aesthetic of the IOTN‐AC; IOTN AC ‐ E, professional aesthetic perception of the IOTN‐AC.

^a^ Friedman test followed by Durbin‐Conover analysis.

^b^ Mann–Whitney *U* test.

In the OQLQ and OHIP‐14 indices, higher scores indicate a greater negative impact on OHRQoL. Both the CA and FA groups showed a progressive improvement in OHRQoL scores throughout the treatment period, as measured by the OQLQ indices. Significant improvements were observed in both groups at T2 and T3, following appliance installation. The intragroup analysis of OHIP‐14 scores showed significant improvements only in the CA group over time. Despite these improvements, no statistically significant differences were found between CA and FA groups at baseline or after 6 months of treatment (Table 2).

The OQLQ domains assessed facial aesthetics, oral function, aesthetic awareness and social aspects. There were no significant differences between groups 1 week after orthodontic appliance installation (T1) For both groups, facial aesthetics had a significant improvement 4 weeks post‐surgery (T2), with no difference 6 months after surgery (T3). For both groups, the oral function improved significantly only in T3. Aesthetic awareness had a significant improvement only in T2 in the FA group and had a significant improvement in T2 and T3 in CA group. The social aspects had a significant improvement in T2 and T3 in both groups. Additionally, no significant differences were found between the two groups across the four OQLQ domains, both at baseline and after 6 months (*p* > 0.05) (Table 3).

**TABLE 3:** Comparative statistics involving the four domains of the OQLQ intragroup in patients using clear aligners and fixed appliances.

OQLQ domains		T0 median (Q1–Q3)	T1 median (Q1–Q3)	*p* ^a^	T2 median (Q1–Q3)	*p* ^a^ (T1–T2)	T3 median (Q1–Q3)	*p* ^a^ (T2–T3)
				(T0–T1)				
Facial aesthetics	FA	18 (17–19)	18 (17–20)	1	7 (2–11)	< 0.001	3 (0.2–5)	0.2
	CA	17.5 (13.8–20)	17 (10.5–18)	0.1	4.5 (1.5–10.3)	< 0.001	2 (0–6)	0.08
Oral function	FA	11 (7–14.8)	13.5 (11–16)	0.09	10 (4–16.8)	0.07	2 (0–5.5)	0.001
	CA	11.5 (8.5–13.3)	12 (5.75–15)	1	11.5 (8–14.3)	1	4 (1–6)	0.001
Aesthetic awareness	FA	8 (5–11.8)	8,5 (7–11.8)	0.2	7 (2.2–9.7)	0.05	1.5 (0–6)	0.08
	CA	9.5 (4–13)	11 (7–13.3)	0.2	8 (1–10)	0.006	3 (0–6)	0.02
Social aspects	FA	18.5 (10.5–24.3)	17.5 (13.3–24.8)	0.6	6.5 (1.7–9.7)	0.002	2 (0–4)	0.01
	CA	17.5 (7.7–27)	17.5 (6–55)	0.3	7 (1.7–15)	0.004	2 (0–5.7)	0.002
*p* ^b^		> 0.05	> 0.05		> 0.05		> 0.05	

*Note:* (T0) pre‐treatment, (T1) 1 week after orthodontic appliance installation, (T2) 4 weeks post‐surgery, and (T3) 6 months post‐surgery. *p* ≤ 0.05 = statistically significant.

Abbreviations: CA, clear aligners; FA, fixed appliance.

^a^ Friedman test followed by Durbin‐Conover analysis.

^b^ Mann–Whitney *U* test.

The OHIP‐14 comprises seven domains: functional limitation, physical pain, psychological discomfort, physical disability, psychological disability, social disability and handicap. There were no significant differences between groups 1 week after orthodontic appliance installation (T1). Functional limitations were observed on T2 and T3 in CA group and only in T3 in FA group. Physical pain presented significant decrease only in T3 for the CA group. There were no significant differences between groups along the study for psychological discomfort. Physical disability decreased significantly in T3 in the FA group and decreased significantly in T2 and T3 in the CA group. Social disability and Handicap decreased significantly only in T3 in both groups. No statistically significant differences were found between groups in any of the seven OHIP‐14 domains, either at baseline or after 6 months of treatment (*p* > 0.05) (Table 4).

**TABLE 4:** Comparative statistics involving the seven domains of the OHIP‐14 intragroup in patients using clear aligners and fixed appliances.

OHIP‐14 domains		T0 median (Q1–Q3)	T1 median (Q1–Q3)	*p* ^a^	T2 median (Q1–Q3)	*p* ^a^ (T1–T2)	T3 median (Q1–Q3)	*p* ^a^ (T2–T3)
				(T0–T1)				
Functional limitation	FA	2 (0–3)	2 (1–4)	0.3	2 (2–3)	0.7	1 (0–1)	0.01
	CA	2 (0–4)	1.5 (0–2.2)	0.1	3 (1–3.5)	0.02	1 (0–2)	0.03
Physical pain	FA	3.5 (0.9–4)	4.5 (3–5.7)	0.1	4.5 (2–5)	0.3	3 (2.7–4)	0.08
	CA	3.5 (0.9–5)	4 (1–4)	0.5	4 (3.5–5)	0.1	3 (1–4)	0.04
Psychological discomfort	FA	3.5 (0.9–4)	4 (2–5.5)	0.8	3 (2–4.7)	0.3	2 (1–2.5)	0.4
	CA	3.5 (0.9–5)	2.5 (1.5–6.2)	0.1	3 (2–3.5)	0.7	2 (0.5–2)	0.1
Physical disability	FA	0.5 (0–1.7)	1.5 (0–4)	0.1	2.5 (1.2–4.7)	0.1	1 (0–2.2)	0.008
	CA	1 (0–2.2)	1 (0–3)	0.4	3 (1.5–4)	0.005	1 (0–2.5)	0.002
Psychological disability	FA	4 (2.2–4.7)	3 (2–4.7)	0.6	3 (0.5–3)	0.5	1 (0–2)	0.04
	CA	4 (1–6)	4 (1–6)	0.5	2 (0.5–3)	0.008	1 (0–2)	0.03
Social disability	FA	1.5 (1–3.7)	1 (0–2.7)	0.3	2.5 (0.2–4)	0.2	0.5 (0–1.2)	0.03
	CA	2 (0–3.2)	2 (0–5)	0.8	2 (1–3)	0.3	1 (0–1)	< 0.001
Handicap	FA	0 (0–1)	0 (0–0.7)	0.3	1.5 (0–3)	0.08	0 (0–1)	0.01
	CA	1 (0–3.2)	1 (0–2)	0.1	1 (0–2)	0.3	0 (0–0)	0.001
*p* ^b^		> 0.05	> 0.05		> 0.05		> 0.05	

*Note:* (T0) pre‐treatment, (T1) 1 week after orthodontic appliance installation, (T2) 4 weeks post‐surgery and (T3) 6 months post‐surgery. *p* ≤ 0.05 = statistically significant.

Abbreviations: CA, clear aligners; FA, fixed appliance.

^a^ Friedman test followed by Durbin‐Conover analysis.

^b^ Mann–Whitney *U* test.

SF‐36 assesses multiple domains of physical and emotional health. There were no significant differences between groups 1 week after orthodontic appliance installation (T1). For both groups, the physical functioning improved significantly only in T3. The role physical decreased significantly from T1 to T2 and increased significantly from T2 to T3. Bodily pain increased significantly only in CA group in T2 and decreased significantly in T3. For this score, there was no difference among the four timepoints, in the FA group. General health improved significantly in both groups in T2. Social functioning improved significantly in both groups only in T3. Role emotional improved only in CA group in T3. Vitality and mental health had no significant difference between groups along the study. No statistically significant differences were found between FA and CA groups (*p* > 0.05) (Table 5).

**TABLE 5:** Comparative statistics involving the eight domains of the SF‐36 intragroup in patients using clear aligners and fixed appliances.

SF‐36 domains		T0 median (Q1–Q3)	T1 median (Q1–Q3)	*p* ^a^	T2 Median (Q1–Q3)	*p* ^a^ (T1–T2)	T3 median (Q1–Q3)	*p* ^a^ (T2–T3)
				(T0–T1)				
Physical functioning	FA	97.5 (76.3–100)	100 (81.3–100)	0.9	80 (67.5–100)	0.08	100 (92.5–100)	0.002
	CA	95 (90–100)	95 (80–100)	0.1	80 (71.3–90)	0.2	100 (90–100)	0.002
Role physical	FA	100 (75–100)	100 (75–100)	0.8	0 (0–75)	0.008	100 (62.5–100)	0.01
	CA	100 (75–100)	100 (75–100)	0.9	12,5 (0–93.8)	< 0.001	100 (100–100)	< 0.001
Bodily pain	FA	62 (61–81)	67 (43.8–84)	1	74 (64.5–83)	0.7	84 (72–100)	0.2
	CA	84 (69.5–85.8)	84 (64.5–100)	0.3	67 (51.3–84)	0.03	84 (74–100)	0.005
General health	FA	59.5 (48.3–67)	54.5 (47–67)	0.3	67 (58.3–72)	0.04	62 (52–75)	0.5
	CA	57 (50.8–73.3)	62 (52–67)	0.1	72 (62–74.3)	0.01	72 (64.5–80)	0.2
Vitality	FA	52.5 (46.3–60)	55 (51.3–63.8)	0.06	57,5 (55–63.8)	0.6	60 (50–65)	0.6
	CA	55 (48.8–61.3)	55 (50–60)	0.5	55 (51.3–60)	0.8	55 (42.5–57.5)	0.2
Social functioning	FA	81.3 (65.6–96.9)	62.5 (40.6–96.9)	0.1	50 (37.5–62.5)	0.9	75 (50–100)	0.04
	CA	75 (62.5–100)	68.8 (50–87.5)	0.3	62,5 (37.5–87.5)	0.3	87.5 (75–100)	< 0.001
Role emotional	FA	100 (41.3–100)	83 (8.25–100)	0.1	66 (33–100)	0.9	83 (0–100)	0.49
	CA	66 (24.8–74.5)	66 (33–100)	0.5	33 (0–100)	0.2	100 (66–100)	0.005
Mental health	FA	80 (53–80)	72 (60–76)	0.2	64 (54–87)	0.1	76 (56–82)	0.7
	CA	68 (35–72)	64 (49–76)	0.2	76 (61–84)	0.07	80 (66–88)	0.3
*p* ^b^		> 0.05	> 0.05		> 0.05		> 0.05	

*Note:* (T0) pre‐treatment, (T1) 1 week after orthodontic appliance installation, (T2) 4 weeks post‐surgery and (T3) 6 months post‐surgery. *p* ≤ 0.05 = statistically significant.

Abbreviations: CA, clear aligners; FA, fixed appliance.

^a^ Friedman test followed by Durbin‐Conover analysis.

^b^ Mann–Whitney *U* test.

## 4 Discussion

This study evaluated the impact of QoL on overall health using the OQLQ, OHIP‐14 and SF‐36 questionnaires, all validated in Portuguese. OHIP‐14 is widely used for assessing OHRQoL [25, 26], but was not specifically designed for orthognathic surgery, whereas OQLQ was developed for this purpose [27], making it a valuable complement. Although SF‐36 is not limited to orofacial conditions, it remains a recognised tool for evaluating general health [23], making it relevant for orthognathic surgery patients [23]. To our knowledge, this is the first study to assess both OHRQoL and psychological outcomes using SF‐36 in aligner‐treated patients undergoing orthognathic surgery.

Despite high‐quality prospective studies on the impact of orthodontic treatment on OHRQoL are limited [16, 17], existing literature suggests significant improvements regardless of the appliance used [28]. This study found that OQLQ and OHIP‐14 scores improved in both CA and FA groups, aligning with previous research. Alfawal et al. [29] and Leyva et al. [16] also reported enhanced QoL in both groups after treatment. Improvements, in the present study, were most evident 6 months after treatment initiation, consistent with studies showing progressive score enhancement. Pelo et al. [27] observed a decline in QoL during the pre‐surgical phase, followed by rapid post‐surgical recovery, while Gao et al. [7] found that pain and anxiety peaked on the first day of treatment but gradually declined in both groups.

When analysing the domains of each group independently, a progressive improvement in QoL was observed across all OQLQ domains. The most significant gains in facial aesthetics and social aspects occurred within the first month post‐surgery. In contrast, improvements in oral function and aesthetic awareness were more gradual, with notable progress observed between 1‐ and 6‐month post‐surgery. High patient satisfaction after orthognathic surgery has been linked to improvements in appearance, function, comfort and self‐confidence [30, 31]. Cordeiro et al. [31] reported significant QoL enhancements in all OQLQ domains over a two‐year period, while Johanson et al. [32] observed similar progress in follow‐ups of up to 24 months, though both studies focused only on FA tretaments. In contrast, Leyva et al. [16] found superior QoL outcomes with CA, but their study did not analyse individual questionnaire domains, limiting a detailed understanding of specific improvements.

OHIP‐14 domains showed that both groups exhibited progressive improvements in psychological domains of the OHIP‐14. The CA group showed significant intra‐group improvements in physical pain between T2 and T3, although, the final scores were comparable between groups. Even that CA group had worse results between T1‐T2 on physical disability and psychological disability, both groups demonstrated significant improvements in these scores over time. These findings suggest that both treatment modalities contribute to psychological well‐being throughout the postoperative period. Previous studies highlighting aligners as a more comfortable and less invasive option compared to FA [7, 33]. Antonio‐Zancajo et al. [33] reported that aligner users experienced less chewing discomfort and lower psychological and social disability during the first week of treatment. Similarly, Gao et al. [7] and Alfawal et al. [29] found better short‐term OHIP‐14 scores with aligners in non‐surgical cases.

SF‐36 results showed that both CA and FA significantly improved patients' psychosocial well‐being, with the most notable progress occurring 6 months after surgery. Although, no statistically significant differences were observed between groups at T3, the CA group demonstrated a significant intra‐group improvement in the Role emotional domain between T2 and T3 (*p* = 0.005). This positive trend may be associated with factors such as greater aesthetic acceptance and the more discreet nature of aligners, potentially contributing to a favourable psychological adaptation throughout the treatment. Both groups experienced an initial decline in physical and general health scores during the first month, likely due to post‐surgical discomfort and treatment adaptation. However, these scores gradually improved over time, in line with previous studies reporting similar short‐term setbacks during the early stages of orthodontic‐surgical treatment as occlusal adjustments and appliance adaptation occur [29].

Although, the CA group showed a temporary increase in pain between T1 and T2, followed by significant improvement at 6 months. This contrasts with previous studies reporting more prolonged pain with FA [34, 35], often linked to wire adjustments. According to Chan et al. [35], this additional orthodontic stimulus can prolong the perception of pain for more than 7 days, unlike appointments where no wire changes occur. In this study, FA patients may have experienced less initial discomfort due to the use of non‐activated archwires, while CA patients may have perceived greater initial pain from the insertion and removal of aligners, despite the absence of attachments. Moreover, while CA is often considered more comfortable, some studies suggest it may not always yield better outcomes. Alajmi et al. [36] reported temporary speech disturbances with CA, and a systematic review found that FA patients had better OHRQoL at the end of treatment [13]. These findings may reflect case selection biases [37], as OHRQoL can be influenced by factors such as age, extractions [15], and socioeconomic status [38]. In contrast to prior studies, the present study controlled for these variables by maintaining a defined age range, excluding extraction cases, and ensuring group homogeneity, thereby minimising potential confounding factors. Interestingly, this study found no negative impact on QoL in either group during the first week of treatment, differing from previous reports of early OHRQoL decline due to appliance‐related discomfort [29, 39]. This may be explained by the use of aligners without attachments in the CA group and non‐activated archwires in the FA group, both contributing to smoother initial adaptation. However, at T2, post‐surgical factors such as pain, swelling and functional limitations likely led to a temporary decline in QoL.

It is worth mentioning that this study has some limitations, including the absence of randomization. Patients were consecutively allocated based on treatment availability, due to practical and ethical constraints. To minimise selection bias, strict eligibility criteria were applied, and baseline assessments confirmed group homogeneity in skeletal severity (Wits appraisal), treatment complexity (IGC), and aesthetic impairment (IOTN AC‐E), supporting their comparability for statistical analysis. Importantly, the numerical difference between groups did not significantly affect the validity of intra‐ or intergroup comparisons. Although the sample size was adequate for detecting the primary outcome, it may have limited the identification of subtle differences. The single‐center design and strict eligibility criteria may reduce generalizability, and the short follow‐up may not reflect long‐term harms or rare adverse events. Future studies with larger, more diverse samples and different malocclusion types are recommended to confirm and expand these findings.

Nevertheless, both CA and FA were effective in improving OHRQoL and patient well‐being in the early phase. This improvement may be attributed to malocclusion correction and patient adaptation to orthodontic treatment [29]. The observed functional improvements in this study were likely due to better appliance adaptability and effective occlusal and facial corrections [26], creating a more favourable environment for function. Similar trends have been observed in other orthodontic treatments, where initial declines in OHRQoL improve as malocclusion is corrected [39]. However, it is suggested that early QoL gains may be influenced by increased aesthetic awareness following surgery [32]. Long‐term follow‐up is essential to determine whether these improvements are maintained [40]. Supporting this, Cordeiro et al. [31] reported a decline in OHRQoL among patients still undergoing orthodontic treatment 2 years after surgery.

Therefore, caution is needed when extrapolating these findings to the broader population of orthognathic surgery patients, as the initial aesthetic improvement may overshadow other concerns that remain underdiagnosed [31]. Given the complex interplay of physical and psychological factors in dentofacial deformities and orthodontic‐surgical treatments, subjective elements can significantly influence OHRQoL and should be carefully considered in result interpretation and future research. Our findings contribute to refining clinical approaches, highlighting the importance of comprehensive patient management before and after surgery to optimise treatment outcomes and overall well‐being.

## 5 Conclusions

The results of this study indicate that both clear aligners and fixed appliances, when combined with the Surgery‐First Approach in Class III skeletal malocclusion patients, significantly enhance Oral Health‐Related Quality of Life and overall well‐being within the first 6 months of treatment. These findings confirm that such improvements occur regardless of the orthodontic appliance used.

## Author Contributions

**Arthur Cunha and José Augusto Mendes Miguel:** conceptualisation and methodology. **Arthur Cunha:** software, data curation, writing – original draft and preparation. **Henrique Martins da Silveira and José Augusto Mendes Miguel:** validation, writing – review and editing, supervision and project administration. **Luana Karine Amaro Silva:** formal analysis. **Arthur Cunha and Luana Karine Amaro Silva:** investigation. All authors have read and agreed to the published version of the manuscript.

## Ethics Statement

The study was approved by the Research Ethics Committee of Pedro Ernesto University Hospital, affiliated with the State University of Rio de Janeiro (UERJ), under protocol number (CEP/HUPE: 51444621.0.0000.5259).

## Conflicts of Interest

The authors declare no conflicts of interest.

## Data Availability

We confirm that the data will be made available in accordance with the journal's policies and preferences. We are committed to complying with the guidelines to ensure proper data sharing and transparency.
